# Supplementary figures and images for: Early biochemical analysis of COVID-19 patients helps severity prediction
Source: PLoS One. 2023 May 19;18(5):e0283469. doi: 10.1371/journal.pone.0283469 (PMC10198541; doi:10.1371/journal.pone.0283469)

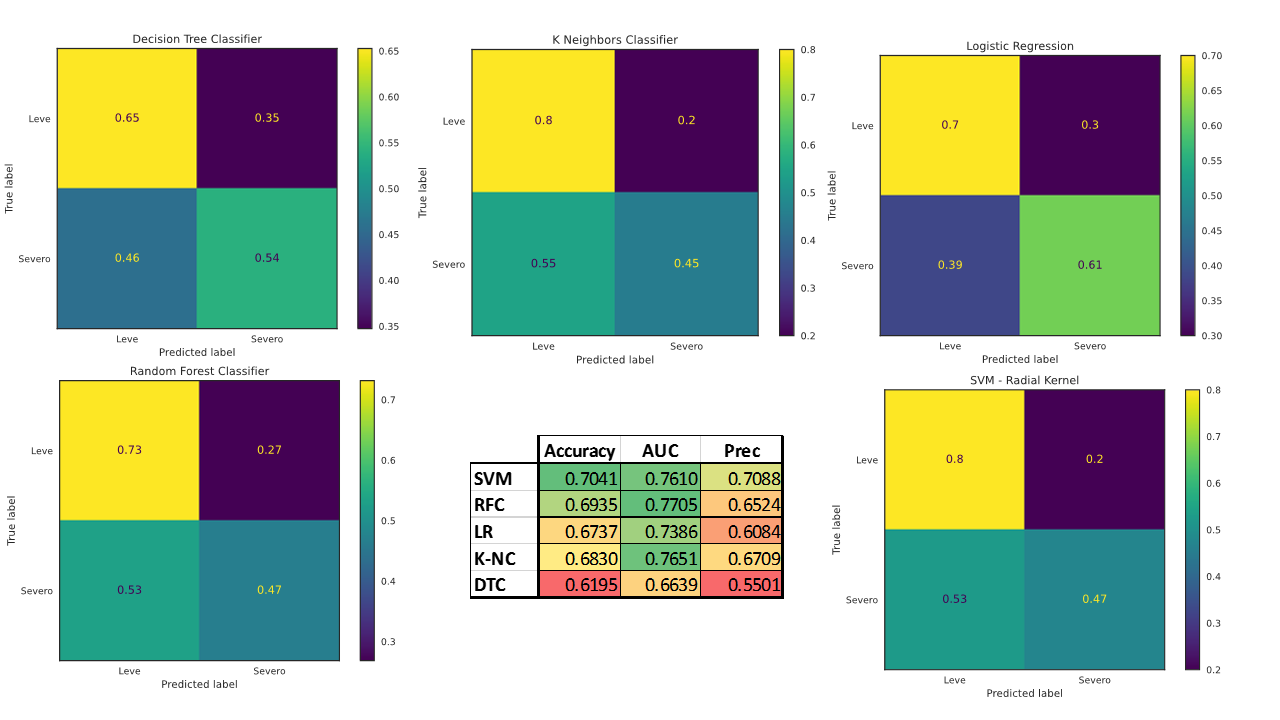

Supplement: S1 File — (ZIP) [file pone.0283469.s001.zip › Supp information/Supp Fig 1.tif]
