# Supplementary material for: Early biochemical analysis of COVID-19 patients helps severity prediction
Source: PLoS One. 2023 May 19;18(5):e0283469. doi: 10.1371/journal.pone.0283469 (PMC10198541; doi:10.1371/journal.pone.0283469)
Supplement: Attachment — Submitted filename: response to reviewers_PLos One v 3.docx [file pone.0283469.s002.docx]

**RESPONSE TO REVIEWERS**

Dear Dr. Arteaga-Livias

Thanks for the opportunity to answer the reviewer’s comments. We are grateful for their carefully reading of our work and for their helpful advices and comments. We answer point-by-point each comment ad rewrite the manuscript incorporating these changes. We make an effort in explain better the algorithm and the used methodology an also the novelty and the focus of this work

**REVIEWER 1:**

**Reviewer #1: Overall, the study was very well conducted. I start by congratulating the authors.**

We thank the reviewer for their comment and their help to improve the manuscript

***Below are my comments and I hope they are useful to improve your study.
1. Line 63, “Continuous variables have been described by the mean, minimum and maximum data”. Clinical data have almost always been shown not to have a normal distribution, my suggestion is that these results be presented in the form of median and interquartile range.***

We totally agree with the reviewer´s suggestion, data presentation has been changed in the new manuscript version.

***2. Line 73, “Statistical significance was defined as follows: p<0.001***”. Why are the levels of significance different? Was there any sampling procedure that was done to get to this point?***

Our impression is that there is some misunderstanding. The type I level of significance has been set at 0.05, however in the charts, one star means that the p-value is below 0.05, two stars mean that is below 0.01, and three stars that is below 0.001. We will rewrite it in the new manuscript version.

***3.On the lines 99-100, "The Model Training step is 100 described in Figure 1". I saw to figure 1, it's actually just a graph of the age distribution as a function of severity. I understood nothing. Are you sure it's figure 1?***

Sorry about this mistake, as you notice there is an error in the order of the uploaded figures. Figure 1 is indeed a schematic description of the model.

***4.Lines 100-101, “Finally, a Cross-Validation 102 method is applied to decrease overfitting impact”. What type of cross-validation was used? Venetian blind?, leave-one-out?***

We use k-fold as a cross-validation method. This procedure has been used with a k =10 meaning a 10-fold cross validation. This information has been added to the new version of the manuscript.
***5.I encourage the authors to insert the formulas for calculating the metrics for evaluating the performance of the models: sensitivity, specificity, accuracy (material and methods). Still on the evaluation of the performance of the models, I ask that the authors discuss the following scientific articles: PMID: 35751188, PMID: 34091385.***

The formulas used are the ones by default in the used approach (SciPy):

- Sensitivity: True positives /(True positives+ False Negatives)

- Specificity: True Negative / (True negative + False Positives)

- accuracy: (True positive + True Negative) / (True Positive + True Negative + False Positive + False Negative)

We include in the discussion the proposed references.

***6.Still on the evaluation of the performance of the models, I ask that authors present a table of confusion matrix with: true positives, true negatives, false positives, false negatives, sensitivity, specificity and accuracy. This must be shown for all classifiers used. For more understanding, see the PMID paper table: 35751188.***

A new supplementary figure including the Table with the values for each model has been generated and added as Supplementary information.

***7.What types of preprocessing are used for machine learning data? I encourage authors to clarify, in the material and methods section and in the Results section.***

The dataset was constructed with the 42 features of 1082 patients, from these 37 variables were chosen avoiding redundancy. Aassociation and correlation between the variables were analyzed (AutoDiscovery Strength Score) to avoid model overfitting and to identify the patient's features that were more associated with the predictive goal. In this specific case, the association of the variables with the severity variable was evaluated and one of the correlated pair of variables was removed, if any. To do that, AutoDiscovery selected the proper numerical method based on the data type and distribution of the variables assessed. Numerical tests applied in each case are Spearman’s Rank Correlation, Variance Analysis (ANOVA one-way, U Mann-Whitney, and Kruskal-Wallis; normality was tested in these cases with D’Agostino/Pearson methods), and Cramer’s V Contingency Index. This massive data exploration process was carried out systematically for each patient group (stratum), considering only those correlations evaluated in a data subset with n≥5, two-tailed p < 0.05 and Spearman’s rank correlation coefficient > 0.8 to be relevant.

Given the nature of this multiple testing method, a high significance threshold was calculated based on Benjamini-Hochberg method (False-Discovery Rate) to classify the rest associations.

The Feature Selection process, where, based on Recursive Feature Elimination (RFE) and the AutoDiscovery Strength Score, age decade and the Lymphocytes to Immature Granulocyte ratio were added as variables for the model.

This information has been added to the manuscript.

**REVIEWER 2:**

***The article needs revision and re-writing with good language and grammar.***

Thanks to the reviewer for his/her comments that help us to improve the manuscript

***1. What is the novelty of this research?***

Although research in COVID19 moves very fast and the needs are changing, our work present6 an algorithm based in data that are obtained in all Hospitals routinely and no need for any extra analysis. That is novel and helpful for all the hospitals in the different countries to take fast decisions. Moreover our work underline the importance of the immature granulocites status in the gravity of the evolution.

***2.What did the authors find new ?***

We present an algorithm based in routinely parameters obtained in the clinical routine. Therefore decisions can be made in the first days of hospitalization without any additional analysis, just based in the regular data obtained the first day of hospitalization. We also highlight the importance of immature granulocytes in the evolution of the disease.

***3. The data is old and dates back to the year 2020, and much research has been published on this topic.***

We partially agree with the reviewer. We try to share our data as quickly as we could (and for that reason manuscript was located at BioRiv repository), however although the data are from the 2020, this is the most naïve data (without vaccinated people) and the ones that give us a more accurate idea about how the disease work. That could be useful for a new coronavirus pandemic and also to understand better the new cases that re arriving to the hospitals.

***4. Why did the authors not focus on albumin levels, despite the fact that albumin was found to decrease in much of the research.***

The aim of the work was not to select the biomarkers but to use the ones that are routinely produced in the regular clinical practice. In fact we recover the data from the clinical records with no interaction (at that moment) with the clinicians, meaning that the choice of variables was based in the clinical needs and in the regular protocols. Is an undirected approach with the aim to find an easy and cheap way to make decisions at the hospital.

***5.Why did the authors not focus on LDH levels, despite the fact that albumin was found to increase in much of the research.***

Please, see response to answer 4.
***6.Most  of the discussion is repetition of the result. Please delete and explore the result through analysis of the result***

Thanks to the reviewer for his/her suggestions. We rewrite the discussion focusing in the analysis of the results.
