# Supplementary material for: Early biochemical analysis of COVID-19 patients helps severity prediction
Source: PLoS One. 2023 May 19;18(5):e0283469. doi: 10.1371/journal.pone.0283469 (PMC10198541; doi:10.1371/journal.pone.0283469)
Supplement: Attachment — Submitted filename: response to reviewers 2nd round.docx [file pone.0283469.s003.docx]

**RESPONSE TO REVIEWERS**

Dear Dr. Arteaga-Livias

Thanks for the opportunity to answer this second round of reviewer’s comments. We are grateful for their carefully reading of our work and for their helpful advices and comments. We answer point-by-point each comment ad rewrite the manuscript incorporating these changes.

**REVIEWER 1:**

**Dear Editor, I would like to thank you for the opportunity to review the article “Early biochemical analysis of COVID-19 patients helps severity prediction”, and I would like to take this opportunity to apologize for the delay in completing the review, I had unforeseen personal problems. Second, I would like to congratulate the authors for the article. Overall I enjoyed the post however some improvements need to be made. My observations follow.**

**1.In the “Abstract” section, the authors describe “…The test has been done by standardized cost-effective technique available in all the hospitals”. If the method is being used in clinical practice, it means that the model has been deployed. If so, I strongly recommend that you provide the access link for your Machine Learning App. I am just saying that the authors need to be realistic, because if the App has not been developed for the developed models, I recommend that they remove the phrase “…The test has been done by standardized cost-effective technique available in all the hospitals” in the manuscript.**

Sorry for the misunderstanding. The model has been developed from biochemical data obtained in a routine assay. This sentence wants to emphasize that this kind of data could be easily obtained in the vast majority of hospitals and therefore the methodology proposed in our work can be easily transferred to clinical practice. We change this phrase in the abstract to clarify the point.

**2.In the material and methods section, “Continuous variables have been described by the mean, minimum and maximum data…”. I suggest initially testing the normality of the data. If normality is proven, the results must be presented as mean and standard deviation, otherwise the results must be presented as median and interquartile range.**

Thanks for the suggestion. As requested by a reviewer we change the presentation of these data to mean, minimum and maximum because, according to the reviewer, is the way in which clinical data should be presented.

**3.The data used in this study are unbalanced: severe cases 449 (41.5%); ICU admission 60 (5.55%); deceased 140 (12.94%). It is important to point out that for imbalance problems, the AUC is not a better metric to evaluate the performance of the classifiers. The most reliable metric is the “F1 Score”. I strongly recommend that authors report these data in the article, including citing them in the abstract.**

**4.Still in material and methods, the authors describe “…Finally, a Cross-Validation 102 method is applied to decrease overfitting impact”. It needs to be clear which type of cross-validation was adopted.**

**5.The figures are very blurred. I ask you to improve the resolution of the figures. I also ask you to increase the size of the X and Y axes of the figures.**

**REVIEWER 2:**

**The author response to all reviewer comment , and the manuscript developed to the level of publication**
